# Editorial

**Published:** 1873-01

**Authors:** 


### Editorial.

"Out of the fullness of the heart the mouth speaketh"—and thus do we wish our readers a "Happy New Year." While thanking them for the liberal support, both intellectual and material, with which they have sustained the JOURNAL throughout the past year, we desire to give them the assurance that the JOURNAL, at the beginning of this its 30th year of life, is only just reaching its period of full maturity and vigor, and is to-day in a better position to repay to its readers all that they may do in its behalf than ever before.

The past year was commenced in ashes and desolation, but with energy and determination: these, as usual, have triumphed; out of the ashes have grown laurels, out of the desolation have come hosts of friends.

The JOURNAL begins its thirtieth volume, with the promise of a more talented and more energetic corps of collaborateurs than before, to whom it can point with pride as representative men up to the front rank in their respective departments of science.

The JOURNAL will continue to be, as heretofore, the organ of no party nor clique, but the independent exponent of truth, whatever be its source. Its pages are open to the free and candid expression of opinion from legitimate sources, and while it will in no case descend to personalities, it will boldly denounce shams and charlatanism, however respectably garbed.

It is intended to be the mouth-piece of Rational Medicine, and the organ of its followers throughout the medical world. H.

***Private Retreat for Insane Females.***

Andrew McFarland, M.D., so long and favorably known as Superintendent of the Illinois, and other public hospitals for the insane, proposes to supply a want which has long been felt by both practitioners and the public. We commend his circular to general attention.

OAK LAWN — A PRIVATE RETREAT FOR FEMALE PATIENTS AFFLICTED WITH MENTAL AND NERVOUS DISEASES.

This institution has been founded to meet the wants of the large and increasing class of patients for whom the overgrown State institutions of the country afford no proper provision, and whose cases demand quiet and retirement as absolutely essential to successful treatment. Its intent is to make the surroundings of its inmates as nearly as possible those of ordinary domestic life; to afford a *home*, to which the most sensitive may resort without thereby feeling that anything is forfeited of the respect and valuation of friends and associates,—a feeling which constitutes the chief element of dread in a commitment to the ordinary insane asylum.

The site fortunately obtained is admirably adapted for the purpose. It is situated in the suburbs of the city of Jacksonville, Ill., and about one mile nearly due south of the junction depots of the Toledo, Wabash and Western; Chicago and St. Louis; Peoria and Jacksonville; and Jacksonville N. W. and S. E. railroads. The basis of its extensive grounds was a natural forest of oaks, walnuts, elms, and other deciduous trees, now reduced, by many years of labor and study, and a large expenditure for foreign evergreens and exotic shrubbery, to a scene of quiet beauty to which the eye turns with pleasure in every direction.

Artificial walks and drives make the grounds an agreeable resort at all seasons of the year.

A gate-lodge, of neat architecture, ushers the visitor into the grounds, from whence the present mansion is reached by a winding avenue. It is proposed—as the patronage of the institution may warrant—to dot the grounds over with tastefully-designed cottage residences, where patients, either singly or in limited numbers, with nurses ever present, may enjoy any degree of elegance which their tastes or the desire of their friends may elect. By this arrangement, every luxury of the most refined home can be kept within the patient's reach,—a privilege quite impossible in the classifications of an ordinary hospital.

This design—the growth of twenty-five years of practical experience of the proprietor—is the result of a deeply-settled conviction that the present system of domiciliating the insane is essentially false in principle, and only adapted, if at all, to the maniacal and ungovernable; that perfection is only reached where the transition from the natural home is least felt; that insanity gathers fresh terrors in the mind of its timid subject from the many-storied, awe-inspiring edifice; the jostling crowd of those that reflect and gather new suffering from each other's faces; and the depressing effect of disagreeable associations from which there is no escape.

The design of Oak Lawn will especially commend itself to those partially developed cases of mental disease, where retirement and avoidance of the claims and restraints of society is the want most felt; and, also, to cases in their earliest stage, where prompt treatment may avoid the wide-spread exposures that commitment to the ordinary hospital involves.

The institution will also be a welcome resort to the large class suffering under complaints peculiar to the sex,—complaints in the great majority of instances due simply to impaired or irregularly distributed nerve-force, but which naturally develop insanity unless understood and relieved; also to those afflicted with hysteria, and its train of kindred disorders, so distressing to their subjects, and so little controllable by medical art under ordinary circumstances.

Persons whom ill health, or other causes, may have led to excessive indulgence in narcotics or stimulants, will receive special scientific treatment, by which the morbid appetite can be tempered or overcome.

Paralytic persons, and those whose mental disorder is the result of advanced age, will have apartments and conveniences especially adapted to their needs, and nurses of tried experience and faithfulness.

The proprietor begs respectfully to commend his design to the thoughtful consideration of all reflecting minds, as an advance step in the treatment of the most distressing of maladies. An enlightened public sentiment, directed towards the problem of how best to provide for the insane—now so much discussed in this country and in Europe, will be the best promoter of its success.

***Skin Grafting.***

From the *Medical Record* we learn that Dr. M. Donnelly, of New York City, and a member of the class of 1868-69, of Rush Medical College, has written a very complete paper on "skin grafting," as studied by him in St. Vincent's Hospital; it is specially to be commended for its attention to details. He closes with a summary of principal points, as follows: "1. Rapidity of cure by grafting over other methods. 2. The elasticity and strength of the new skin over ordinary cicatrices. 3. Absence of contraction and deformity. 4. The simplicity of the operation, and its freedom from pain. 5. Necessities: Absolute rest, and healthy granulations. 6. Size of grafts: A quarter of an inch square; smaller almost useless; larger ones unnecessary. 7. The outside of the arm the best place to take the integument from. 8. In large ulcers, the graft to be placed one inch from the edges; the operation repeated as often as required. 9. The full depth of the *true skin* necessary, as growth of grafts is due to the derma, and not to the epidermis."

***A Veteran.***

The *New York Observer*, having completed its fiftieth year as the leader of the Religious Newspaper Press, has prepared for its thousands of subscribers a New Year's Gift in the shape of a JUBILEE YEAR-BOOK, embellished with several appropriate illustrations. The *Observer* was launched in 1823, and for fifty years has sailed in an undeviating course, without once changing its motto or striking its colors. Few papers can present such a successful history; and while there are plenty of good papers published, there are few that we can recommend as strongly as the *Observer* for all the purposes of a family newspaper. Large, comprehensive, and well filled, it cannot fail to pay those who take it four-fold for their outlay. All subscribers get the *Jubilee Year-Book, gratis.* $3 a year. Sidney E. Morse & Co., 37 Park Row, New York.

***The Concours,***

To fill the positions of instructors during the annual spring and summer session of Rush Medical College, has been conducted to a highly successful termination. A goodly number of gentlemen presented themselves as applicants for the several positions, and although all of course could not expect to succeed, each, we may say, without an exception, proved himself amply competent and apt to teach.

In several of the departments, very considerable difficulty was experienced in deciding upon the relative merits of the candidates. Were it proper, we should be happy to mention personally, several whose trial lectures were particularly excellent, although not crowned by an appointment.

The Concours resulted in the following appointments, which are detailed from memory, and not in the order of their occurrence:

Charles T. Parkes, M.D., Anatomy.
F. L. Wadsworth, M.D., Physiology.
P. S. Hayes, M.D., Chemico-Physics.
John E. Owens, M.D., Surgery.
O. B. Adams, M.D., Materia Medica and Therapeutics.
Isaac N. Danforth, M.D., Pathology.
Norman Bridge, M.D., Principles and Practice of Medicine.
Walter Hay, M.D., Diseases of the Mind and Nervous System.
James N. Hyde, M.D., Syphilis.
A. Reeves Jackson, M.D., Diseases of Women and Children.
Philip Adolphus, M.D., Obstetrics.
L. W. Case, M.D., Chemistry.

### *Appointment.*

The Senior Editor takes especial pleasure in chronicling the fact, that his associate, Walter Hay, M.D., has been appointed Adjunct Professor of Principles and Practice of Medicine in Rush Medical College. For some time past, Dr. Hay has given the lectures on Diseases of the Mind and Nervous System. It is sufficient encomium to say, that the medical class heartily applaud the appointment.

### *Statistics of Vaccination.*

Attention is directed to the tables on pp. 56, 57 of the Journal which we are permitted to publish through the kindness of our friend, the able and philanthropic Prof. H. A. Johnson, M.D., member of the Executive Committee of the Board of Relief. Further observations hereafter.

